# Quantitative data describing the impact of the flavonol rutin on in-vivo blood-glucose and fluid-intake profiles, and survival of human-amylin transgenic mice

**DOI:** 10.1016/j.dib.2016.11.077

**Published:** 2016-11-29

**Authors:** Jacqueline F. Aitken, Kerry M. Loomes, Isabel Riba-Garcia, Richard D. Unwin, Gordana Prijic, Ashley S. Phillips, Anthony R.J. Phillips, Donghai Wu, Sally D. Poppitt, Ke Ding, Perdita E. Barran, Andrew W. Dowsey, Garth J.S. Cooper

**Affiliations:** a School of Biological Sciences, University of Auckland, New Zealand; b Maurice Wilkins Centre for Molecular Biodiscovery, University of Auckland, New Zealand; c Department of Surgery, Faculty of Medical & Health Sciences, University of Auckland, New Zealand; d Centre for Advanced Discovery and Experimental Therapeutics, CMFT, Manchester Academic Health Sciences Centre, and Institute of Cardiovascular Sciences, Faculty of Biology, Medicine and Health, University of Manchester, UK; e Michael Barber Centre for Collaborative Mass Spectrometry, Manchester Institute of Biotechnology, University of Manchester, UK; f Key Laboratory of Regenerative Biology and Guangdong Provincial Key Laboratory of Stem Cell and Regenerative Medicine, Guangzhou Institute of Biomedicine and Health, Chinese Academy of Sciences, Guangzhou, China; g Joint School of Biological Sciences, Guangzhou Institute of Biomedicine and Health, Guangzhou Medical University, Guangzhou, China; h College of Pharmacy, Jinan University, Guangzhou, China; i School of Social & Community Medicine, Faculty of Health Sciences, University of Bristol, UK

## Abstract

Here we provide data describing the time-course of blood-glucose and fluid-intake profiles of diabetic hemizygous human-amylin (hA) transgenic mice orally treated with rutin, and matched control mice treated with water. We employed “parametric change-point regression analysis” for investigation of differences in time-course profiles between the control and rutin-treatment groups to extract, for each animal, baseline levels of blood glucose and fluid-intake, the change-point time at which blood glucose (diabetes-onset) and fluid-intake (polydipsia-onset) accelerated away from baseline, and the rate of this acceleration. The parametric change-point regression approach applied here allowed a much more accurate determination of the exact time of onset of diabetes than do the standard diagnostic criteria. These data are related to the article entitled “Rutin suppresses human-amylin/hIAPP misfolding and oligomer formation *in-vitro*, and ameliorates diabetes and its impacts in human-amylin/hIAPP transgenic mice” (J.F. Aitken, K.M. Loomes, I. Riba-Garcia, R.D. Unwin, G. Prijic, A.S. Phillips, A.R.J. Phillips, D. Wu, S.D. Poppitt, K. Ding, P.E. Barran, A.W. Dowsey, G.J.S. Cooper. 2016) [1].

**Specifications Table**

**Table:** 

Subject area	*Biology*
More specific subject area	*Type 2 Diabetes, parametric regression analysis*
Type of data	*Figure, Table*
How data were acquired	*Blood glucose measurements (Advantage II, Roche Diagnostics), fluid intake measurement in rutin-treated and control human amylin transgenic mice*
Data format	*Analyzed*
Experimental factors	*Treatment of human amylin transgenic mice with either rutin (0.5 mg/ml in the drinking water) or water from 21 days of age (weaning)*
Experimental features	*Weekly blood glucose and fluid intake measurements*
Data source location	*University of Auckland, Auckland, New Zealand*
Data accessibility	*Data are presented in this article*

**Value of the data**

• These data will be of value to the scientific community working in the area of type-2 diabetes and anti-diabetic therapies since they illustrate the inhibitory effect of a small orally-active molecule on two specific biological parameters reflecting the progression of diabetes in a model that closely mirrors the human disease.
• These data will also be of value to biostatisticians working in the area of pre-clinical treatment investigations, as they present a new Bayesian statistical approach for analysing the effect of drug treatment on a key diagnostic criterion employed for diagnosis of diabetes in patients and diabetic animals.
• This new Bayesian approach could find application in the evaluation of future therapies for diabetes or other diseases, which have biological parameters that are analysed for clinically relevant impacts.

## 1 Data

**Fig. 1:**
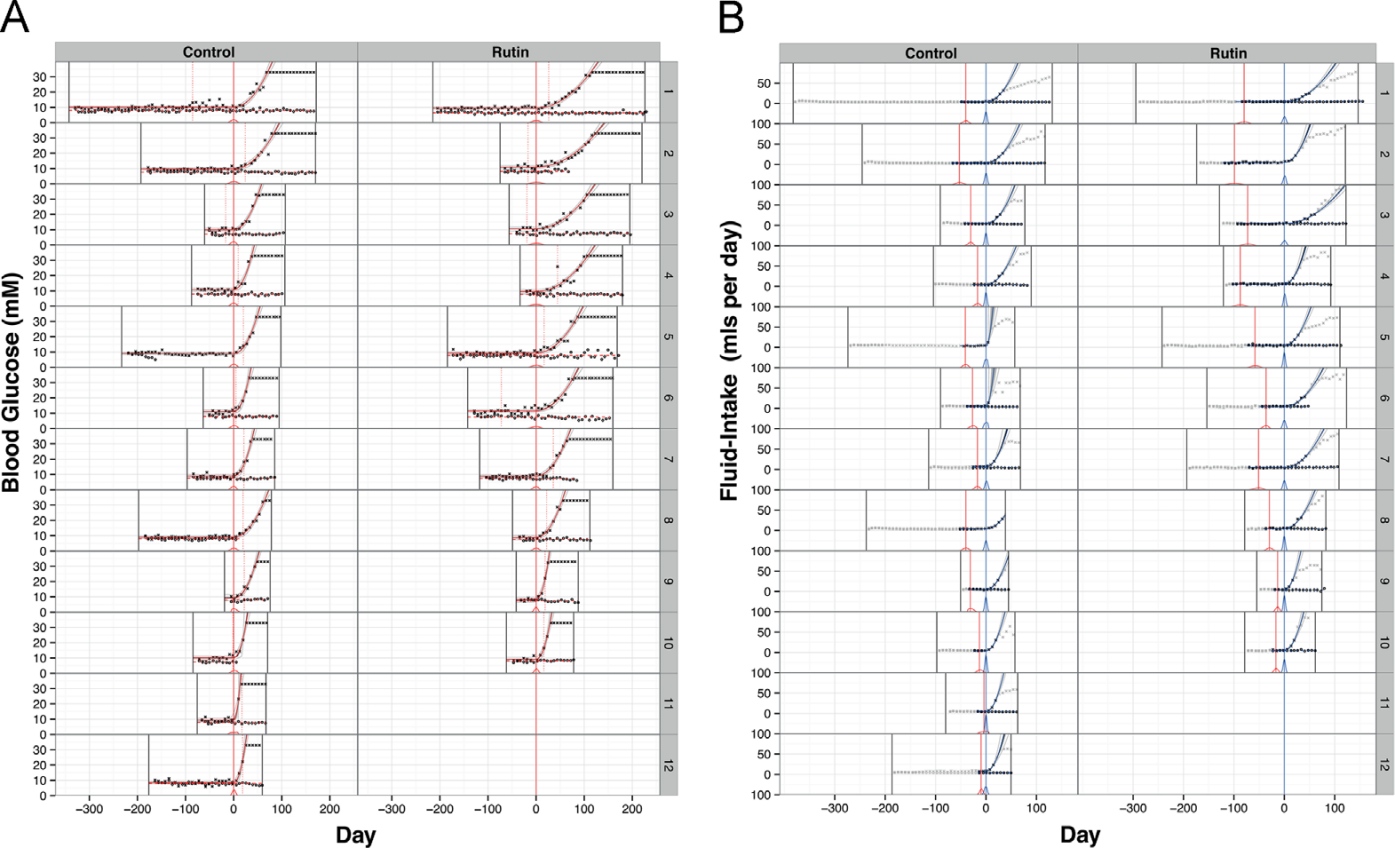
**A.** Blood-glucose profiles of non-transgenic animals (*points*) and transgenic littermates (*crosses*) for the investigation of rutin treatment in hA-transgenic mice (*n*=12 control pairs, *n*=10 rutin-treated pairs). For each pair, the pair׳s weaning and the transgenic׳s day of death are shown as *black-vertical lines*. Each time-course is centred on the transgenic׳s most likely day of diabetes-onset (*red-vertical line*), which was inferred by parametric change-point regression analysis as the time point at which the profile changes from a constant baseline to a constant acceleration from baseline. The most likely fitted profile (joint posterior mode) is shown for each transgenic (*red curve*) and non-transgenic (*dashed-red line*). The uncertainty of the fit is illustrated for each transgenic׳s profile (*grey*, 95% credible interval) and diabetes-onset change-point (*red posterior distribution positioned over the x-axis at day zero*). Results for the conventional method for determining diabetes onset (two consecutive weekly measurements >11 mM) are also shown (*dotted-red vertical lines*), illustrating the inaccuracy and therefore limited utility of this approach for sensitive between-treatment comparisons. Fig. 1**B.** Fluid-intake profiles of non-transgenic animals (*points*) and transgenic littermates (*crosses*) corresponding to the blood-glucose profiles shown in Fig. 1A. Data points removed before parametric modelling are greyed out. Each time-course is centred on the transgenic׳s most likely fluid-intake change-point (*blue-vertical line*), with the respective blood-glucose change-point from Fig. 1A superimposed (*red-vertical line*). The most likely fitted profile (joint-posterior mode) is shown for each transgenic (*blue curve*) and non-transgenic littermate (*dashed-blue line*). The uncertainty of the fit is shown for each transgenic׳s profile (*grey*, 95% credible interval), and the change-points for blood glucose and fluid-intake (*red and blue posterior distributions respectively, both positioned over the x-axis at their respective change-points*). These posterior distributions illustrate that the fluid-intake change-points are estimated with more certainty than the blood-glucose change-points.(For interpretation of the references to colour in this figure legend, the reader is referred to the web version of this article)

Here we present data illustrating the effects of the dietary flavonol rutin on the blood-glucose (see Fig. 1A) and fluid-intake profiles (Fig. 1B) of h-amylin transgenic male mice and their non-transgenic littermates versus mice treated with water (vehicle) only.

We employ a novel parametric change-point regression analysis to extract, for each animal, baseline levels of blood glucose and fluid intake, the change-point time at which blood glucose (diabetes-onset) and fluid intake (onset of polydipsia) accelerated away from baseline, and the rate of this acceleration (Table 1). This enabled more exact measurement of the impact of rutin on the survival of diabetic mice.

## 2 Experimental design and methods

### 2.1 Human-amylin transgenic mice

Protocols were approved by the University of Auckland Animal Ethics Committee and performed in accordance with the New Zealand Animal Welfare Act (1999), the U.K. Animals (Scientific Procedures) Act 1986, and associated guidelines. All protocols complied with the ARRIVE guidelines [2]. The experimental design for rutin versus water treatment of hA transgenic mice and collection of blood glucose and fluid intake data are described in [1].

### 2.2 Modelling of time-dependent glucose and fluid-intake data in rutin- and control-treatment groups

For modelling of time-dependent glucose and fluid intake data in rutin- and control-treatment groups (Fig. 1A,B), we constructed a non-linear parametric change-point regression model, where each of the three inferred parameters had a clear biological interpretation: (i) A constant baseline component; (ii) A change-point in time where blood glucose or fluid intake begins to deviate from this baseline; (iii) A log-transformed constant acceleration from baseline, beginning at the inferred change-point. The maximum likelihood solution to parametric change-point regression in general is a highly non-convex optimisation problem [3], necessitating grid search or similar. To this end, we performed a Bayesian simulation to sample the posterior distribution of the three parameters for each animal, plus a single normally-distributed residual variance parameter across all animals. All parameters were assigned uninformative uniform prior distributions (truncated to between weaning and death for the change-point parameters, and restricted to non-negative values for the variance parameter). The Stan Hamiltonian Monte Carlo software [4] was employed, which efficiently sampled the posterior distribution despite strong correlation between the change-point and acceleration parameters of each animal. Four chains of 2^18^ iterations (of which 2^17^ were warm-up iterations and with thinning factor 2^5^) were generated from over-dispersed starting values. On the result, the Gelman–Rubin convergence diagnostic [5] strongly suggested the posterior was sampled evenly and not affected by local minima (Rhat<1.01 and effective sample size >400 for all parameters). From the generated samples, Fig. 1A, B show the 95% intervals of Highest Posterior Density (HPD) for the predicted time-courses, and the full posterior distributions for the change-point parameters. These indicate predominantly unimodal fit, together with reasonable estimation uncertainty. Finally, from the marginal posterior of each parameter, the median was extracted and used to seed a maximum likelihood fit to the joint posterior mode, as illustrated. The parameter values for this joint posterior mode are presented in Table 1, and were subsequently used for downstream statistical testing.

Glucose measurements were right-censored (at >30.0 mM), which was modelled appropriately by the parametric change-point regression analysis model likelihood.

Modelling of the fluid-intake time-course is shown in Fig. 1B. The fluid-intake time-course had a more complex structure. Firstly, there is typically a transient increase in fluid-intake during adolescence and secondly, fluid-intake in late-stage polydipsia was highly variable between animals. Since the first phenomenon is not directly of interest, we mitigated most of its influence by removing from the analysis all data-points corresponding to times less than the lower HPD boundary of the respective animal׳s inferred blood-glucose change-point. As no inference can be made from the second phenomenon within the current sample size, we removed its effect by right censoring measurements above 30.0 ml/day.

The modelling and inference then proceeded identically (Fig. 1B) to that for the blood-glucose model (Fig. 1A). Inflexion points in longitudinal data were determined by parametric change-point regression analysis, which enabled objective fitting of time-response curves (Fig. 1A, B; Table 1).

## Funding

This research was supported by grants from Endocore Research Trust (60147); Maurice and Phyllis Paykel Trust, New Zealand (various equipment grants; 3601069; co-funding of CD-Spectrometer); Health Research Council of New Zealand, New Zealand (HRC 03/190); Ministry of Business, Innovation & Employment, New Zealand (MBIE; UOAX0815); Maurice Wilkins Centre for Molecular Biodiscovery (Tertiary Education Commission 9431–48507); Lottery Health New Zealand (3354520; co-funding of CD-Spectrometer); Medical Research Council, United Kingdom (UK, MR/L010445/1 and MR/L011093/1); Guangdong High-end Foreign Expert Fund; a Key International Collaborative Fund from Chinese Academy of Sciences (154144KYSB20150019,DW); University of Manchester, the Central Manchester NHS Foundation Trust, and the Northwest Regional Development Agency through a combined programme grant to CADET; and was facilitated by the Manchester Biomedical Research Centre and the Greater Manchester Comprehensive Local Research Network.

## Figures and Tables

**Table 1:** Metadata and parameters inferred from parametric change-point modelling of the control and rutin-treated transgenic mice, together with corresponding results for non-parametric survival analysis (two-tailed Wilcoxon test) and parametric testing (two-tailed *t*-test).

Treatment	ID	**Study metadata**			**Parameters inferred from blood glucose model**					**Parameters inferred from fluid intake model**					**Inferred from both models**
		**Transgenic**			**Transgenic**				**Normal**	**Transgenic**				**Normal**	**Transgenic**
		Wean to Death (Days)	Wean to Diagnosis (Days)	Diagnosis to Death (Days)	Wean to Change-point (Days)	Change-point to Death (Days)	Acceleration from Baseline (log mM^2^/Day)	Baseline (mM)	Baseline (mM)	Wean to Change-point (Days)	Change-point to Death (Days)	Acceleration from Baseline (log mM^2^/Day)	Baseline (ml)	Baseline (ml)	Change-point to change-point (Days)
**Control**	1	514	257	257	342	172	−5.4	10.2	7.9	383	131	−3.7	4.0	3.6	40
	2	363	217	146	193	170	−5.7	9.8	7.6	246	117	−3.8	3.9	3.1	53
	3	168	44	124	61	107	−4.7	9.9	7.1	91	77	−3.5	4.5	3.7	30
	4	194	97	97	88	106	−4.1	10.8	7.8	104	90	−3.6	4.6	4.3	17
	5	331	253	78	233	98	−4.6	9.0	7.6	274	57	−0.7	3.8	–	41
	6	158	68	90	63	95	−3.8	10.9	7.9	90	68	−1.0	5.4	4.0	27
	7	182	118	64	97	85	−4.1	8.9	7.5	114	68	−3.0	7.4	4.4	17
	8	276	217	59	198	78	−5.2	9.0	8.0	238	38	−3.8	4.2	3.7	40
	9	95	41	54	19	76	−4.5	9.1	8.0	50	45	−3.2	4.7	5.0	31
	10	155	83	72	85	70	−3.4	10.1	7.4	98	57	−2.7	4.2	4.4	13
	11	143	93	50	76	67	−2.0	9.5	7.9	80	63	−2.6	4.6	3.9	4
	12	236	195	41	177	59	−3.2	8.5	7.9	186	50	−2.6	8.0	3.6	10
															
**Rutin**	1	441	241	200	215	226	−6.3	9.5	6.5	295	146	−4.7	4.5	4.5	80
	2	295	58	237	75	220	−6.6	10.9	8.2	174	121	−3.4	4.9	4.3	99
	3	251	37	214	56	195	−6.2	10.5	7.4	129	122	−5.0	4.5	4.1	73
	4	213	78	135	33	180	−6.2	9.5	7.7	121	92	−3.0	5.9	5.3	87
	5	353	201	152	184	169	−5.8	9.3	7.7	243	110	−3.4	4.6	4.9	58
	6	277	153	124	117	160	−5.1	8.9	7.3	154	123	−4.1	4.5	4.6	36
	7	302	70	232	142	160	−5.6	11.5	7.4	193	109	−4.2	4.5	5.0	51
	8	161	71	90	49	112	−4.8	8.7	7.5	79	82	−3.7	4.8	4.5	29
	9	129	60	69	41	88	−3.2	8.1	7.5	55	74	−2.4	4.4	3.8	13
	10	140	78	62	62	78	−3.6	9.1	8.1	79	61	−2.8	5.4	4.6	17
**test**		Wilcoxon	Wilcoxon	Wilcoxon	Wilcoxon	Wilcoxon	*t*-test	*t*-test	*t*-test	Wilcoxon	Wilcoxon	*t*-test	*t*-test	*t*-test	Wilcoxon
		**0.582**	**0.222**	**0.044**	**0.228**	**0.011**	**0.030**	**0.932**	**0.276**	**0.974**	**0.014**	**0.059**	**0.744**	**0.009**	**0.036**
**estimate**		**29**	**−24**	**63**	**−27**	**68**	**−1.1**	**0.0**	**−0.2**	**−1.4**	**35**	**−0.8**	**−0.1**	**0.6**	**27.0**
**95% CI lower**		**−63**	**−137**	**0**	**−123**	**9**	**−2.1**	**−0.9**	**−0.6**	**−92.1**	**6**	**−1.6**	**−1.0**	**0.2**	**0.3**
**95% CI upper**		**122**	**26**	**135**	**33**	**109**	**−0.1**	**0.8**	**0.2**	**73.5**	**60**	**0.0**	**0.8**	**1.0**	**54.3**
